# Associations of childhood, maternal and household dietary patterns with childhood stunting in Ethiopia: proposing an alternative and plausible dietary analysis method to dietary diversity scores

**DOI:** 10.1186/s12937-018-0316-3

**Published:** 2018-01-29

**Authors:** Yohannes Adama Melaku, Tiffany K. Gill, Anne W. Taylor, Robert Adams, Zumin Shi, Amare Worku

**Affiliations:** 1 Department of Public Health Sciences, Addis Continental Institute of Public Health, Addis Ababa, Ethiopia; 2 Adelaide Medical School, The University of Adelaide, Adelaide, Australia; 3 Health Observatory, Discipline of Medicine, The Queen Elizabeth Hospital Campus, The University of Adelaide, Adelaide, Australia

**Keywords:** Dietary data analysis, Dietary diversity score, Dietary pattern, Stunting

## Abstract

**Background:**

Identifying dietary patterns that consider the overall eating habits, rather than focusing on individual foods or simple counts of consumed foods, better helps to understand the combined effects of dietary components. Therefore, this study aimed to use dietary patterns, as an alternative method to dietary diversity scores (DDSs), and investigate their associations with childhood stunting in Ethiopia.

**Methods:**

Mothers and their children aged under 5 years (*n* = 3788) were recruited using a two-stage random cluster sampling technique in two regions of Ethiopia. Socio-demographic, dietary and anthropometric data were collected. Dietary intake was assessed using standardized dietary diversity tools. Household, maternal and child DDSs were calculated and dietary patterns were identified by *tetrachoric* (factor) analysis. Multilevel linear and Poisson regression analyses were applied to assess the association of DDSs and dietary patterns with height-for-age z score (HAZ) and stunting, respectively.

**Results:**

The overall prevalence of stunting among children under-five was 38.5% (*n* = 1459). We identified three dietary patterns each, for households (“fish, meat and miscellaneous”, “egg, meat, poultry and legume” and “dairy, vegetable and fruit”), mothers (“plant-based”, “egg, meat, poultry and legume” and “dairy, vegetable and fruit” and children (“grain based”, “egg, meat, poultry and legume” and “dairy, vegetable and fruit”). Children in the third tertile of the household “dairy, vegetable and fruit” pattern had a 0.16 (β = 0.16; 95% CI: 0.02, 0.30) increase in HAZ compared to those in the first tertile. A 0.22 (β = 0.22; 95% CI: 0.06, 0.39) and 0.19 (β = 0.19; 0.04, 0.33) increase in HAZ was found for those in the third tertiles of “dairy, vegetable and fruit” patterns of children 24–59 months and 6–59 months, respectively. Those children in the second (β = −0.17; 95% CI: -0.31, −0.04) and third (β = −0.16; 95% CI: -0.30, −0.02) tertiles of maternal “egg, meat, poultry and legume” pattern had a significantly lower HAZ compared to those in the first tertile. No significant associations between the household and child “egg, meat, poultry and legume” dietary patterns with HAZ and stunting were found. Statistically non-significant associations were found between household, maternal and child DDSs, and HAZ and stunting.

**Conclusion:**

A higher adherence to a “dairy, vegetable and fruit” dietary pattern is associated with increased HAZ and reduced risk of stunting. Dietary pattern analysis methods, using routinely collected dietary data, can be an alternative approach to DDSs in low resource settings, to measure dietary quality and in determining associations of overall dietary intake with stunting.

**Electronic supplementary material:**

The online version of this article (10.1186/s12937-018-0316-3) contains supplementary material, which is available to authorized users.

## Background

Although the global burden of stunting decreased between 1990 and 2015 by more than 25%, it has continued to be a major nutrition-related risk factor causing 257 deaths per 100,000 globally [1]. In sub-Saharan Africa, 11.8 disability-adjusted life years (DALYs) and 136,455 childhood deaths in 2015 were attributable to stunting [2]. In Ethiopia, 38% of children under-five years of age were stunted [3] and it was a risk factor for 960,742 DALYs and 11,065 deaths in 2015 [2]. Stunting also halts the development of societies by negatively affecting mental and physical health [4]. Suboptimal nutrition is a major contributor to stunting in developing countries [5], although there are other causal and contextual factors [4].

Although there are well-established methods, collation and analysis of dietary data have remained challenging in low-income countries (LICs) for various reasons, including high costs, lack of centralized platforms for dietary data, little investment in research, low capacity and technical complexity [6]. As a result, dietary assessment is mainly dependent on approaches which require low cost and provide low quality. Dietary diversity assessment has remained the most commonly used method of data collection, analysis and interpretation approach in LICs. Dietary diversity scores (DDSs) of households, women and children [7–9] are important tools and the most commonly used indicators of assessing the adequacy of nutrient intake. In many studies, it has been also demonstrated that DDSs were useful indicators of micronutrient status [10–13] and a higher DDS is associated with a lower risk of stunting [14–16]. The indicators are relatively simple and suitable for use in large surveys [3]. However, data collected for the purpose of DDS analysis are qualitative, and in most cases, they are dichotomized (yes/no) [8] restricting further analyses. Thus, the analyses depend on a simple count of food groups and do not consider the correlations of the food groups and their impact on nutritional (disease) outcomes. In addition, because the main purpose of DDS analysis is on the number, rather than the type of foods consumed, this may ignore the antagonist, additive and synergistic effect of food groups.

Currently the focus of nutritional epidemiology is to investigate the patterns of multiple food and nutrient intakes without ignoring the interactions. Methodological development over the last two decades enables us to explore the association between diet and disease outcomes through a systematic consideration of the correlation between the components of the overall diet, that is dietary patterns [17, 18]. A study by *Humphries* et al. reported that total food expenditures (using food groups for child DDSs) did not significantly predict HAZ in Ethiopia. In this study, household food group expenditure index, determined by factor analysis of disaggregated food expenditure, was found to be a significant predictor for HAZ [19]. This leads to a premise that a mere aggregate availability and accessibility of the included food groups are not the determining factors for HAZ, rather the specific types of food groups available and their consumption pattern. Another study in the same cohort strengthens this conclusion [20]. Therefore, identifying dietary patterns that consider the overall eating habits, rather than focusing on individual foods (simple counting of consumed foods), better reflect the complexity of dietary intakes and help to understand the combined effect of diet components [21]. In this study, we aimed to identify household, maternal and child dietary patterns and investigate their associations with childhood stunting in Ethiopia using the same dietary data collected for determining DDS. In addition, the study compares the findings with the estimates of associations between DDSs and stunting. To the best of our knowledge, this is the first study investigating the aforementioned objectives.

## Methods

### Study area and participants

A cross-sectional study was conducted in the South Nations, Nationalities and People (SNNP) and Tigray (northern Ethiopia) regions between June and September 2014. The two regions are geographically located at opposite ends of Ethiopia, in the south and north, with differences in agroecology, subsistence farming being the most common occupation in both regions. The SNNP is a larger geographic area and has a greater population size compared to the Tigray region. This study was part of a larger project of the Alive and Thrive’s (A&T) impact evaluation for community-based interventions. The major objectives of the evaluation included assessment of infant and young child feeding (IYCF) practices and stunting prevalence. The baseline and progress evaluation of the project were conducted between June and September 2010 and 2013, respectively.

### Sample size calculation and sampling technique

The sample size was calculated based on the 2010 baseline and 2013 progress evaluation surveys’ estimates. It took into account an intracluster correlation of 0.03–0.04. A total of 75 clusters (enumeration areas [EAs]), a one-sided test, a power of 80%, and a significance level of α = 0.05 were included in the calculations. Based on these considerations, a minimum sample size of 2950 was required (Table 1). However, in total, data were collected from 3788 children and their mothers.

**Table 1:** Sample size calculation, 2014

	Baseline (2010) rate (%)	Expected endline rate (%)	Percentage point (pp) or change in mean z-score	Power	Intra-class correlation at baseline (2010)	Intra-class correlation at 2013 progress evaluation survey	Minimum sample size required
Stunting (24-59 m)	55.9	48.9	7 pp	0.8	0.032	NA	1450
Exclusive breast feeding (0–5.9 m)	72.4	79.4	7 pp	0.8	0.026	0.005	600
Minimum dietary diversity (6–23.9 m)	6.3	11.3	5 pp	0.8	0.038	0.125	900
Minimum total sample size required							2950

**Fig. 1:**
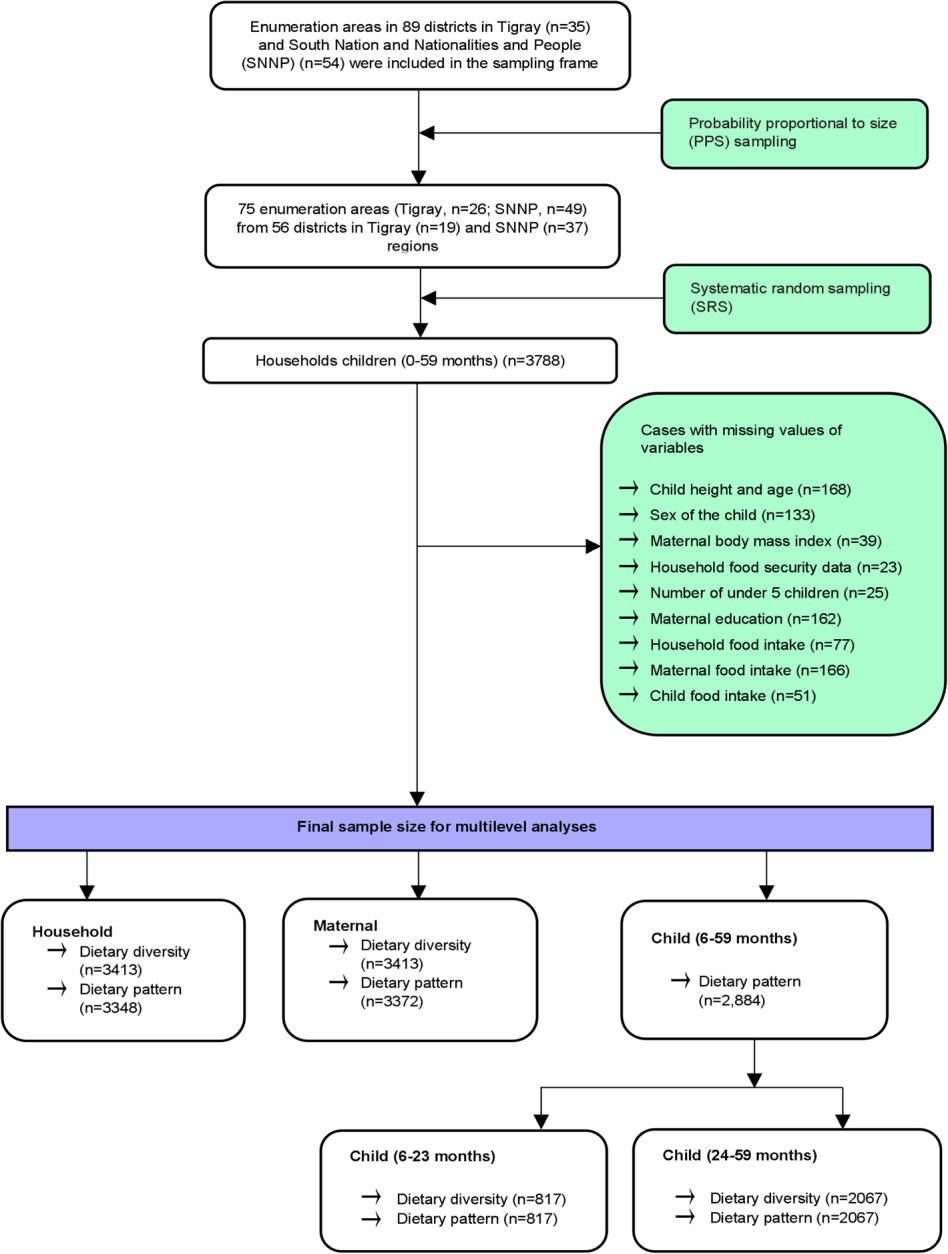
Sample description

A two-stage cluster sampling technique was used to select households with children under five. In the first stage (primary sampling unit), EAs were selected from 89 districts (the second smallest administrative units in Ethiopia). The EA is a geographical unit devised by the Central Statistical Authority (CSA) of Ethiopia, which consists of 150–200 households. This is the smallest cluster used in Demographic and Household Surveys and roughly coincides with the *kebele* (the smallest administrative units) boundaries (Fig. 1). A total of 75 EAs (26 from Tigray and 49 from SNNP), from 56 districts (19 from Tigray and 37 from SNNP) were selected using probability proportional to size (PPS) sampling in relation to the population of the EAs.

In the second stage, children between 0 to 59.9 months (*n* = 3788) were selected. A complete household listing with the number of children residing in each household, in each selected cluster, was developed in collaboration with the local health and administrative offices. This list included identification of all eligible candidates for the survey (mothers of those children under 60 months of age). From this list, three sampling frames were developed: children aged 0–5.9 months, 6–23.9 months, and 24–59.9 months. From each sampling frame, study subjects were selected using a systematic random sampling (SRS). Households selected to participate in one age category were not included in the other sampling frames, even if there were other eligible children in a household.

### Data collection

All data (interview and anthropometric) were collected by trained data collectors who had bachelor degrees or above. To maintain the data quality, demonstrations and pilot testing were conducted during the training period. Trained supervisors oversaw and monitored the data collectors during the field work. The supervisors also checked 5–10% of the anthropometric and interview data to ensure reliability.

### Anthropometry data collection

The length or height of children was measured to the nearest 0.1 cm using the United Nations International Children’s Emergency Fund (UNICEF) recommended wooden board with an upright wooden base and movable headpieces. Children ≥24 months were measured while standing upright while those less than 24 months in the recumbent position. Weight was measured to the nearest 0.1 kg using UNICEF’s scale [22]. Immunization cards or home records of the date of birth, if available, were used to determine the age of the children. In the case of absence of these documents, mother’s recall was taken using the local calendar and then converted to the Gregorian calendar. Adult weighing and height scales were used to measure maternal weight and height, respectively. Mothers were asked to remove shoes and heavy cloths before weight and height were measured. Weight and height were recorded to the nearest 0.1 kg and 1.0 cm, respectively.

### Dietary data collection

Dietary data for children [7] and women [8] and household dietary and food insecurity data [9, 23] were assessed using standard tools. Dietary intake was assessed for the preceding day (24 h). For children aged 6–23 months, seven food groups (grains, roots and tubers; legumes and nuts; dairy products (milk, yogurt, cheese); flesh foods (meat, fish, poultry and liver/organ meats); eggs; vitamin-A rich fruits and vegetables; and other fruits and vegetables) were included [7]. For children 24–59 months, an additional two food groups (oils and fats and independent categories of other fruits and vegetables) were included. The maximum dietary diversity for women of reproductive age (MDD-W) assessment includes 10 food groups (grains, white roots and tubers, and plantains; pulses (beans, peas and lentils); nuts and seeds; dairy; meat, poultry and fish; eggs; dark green leafy vegetables; other vitamin A-rich fruits and vegetables; other vegetables; other fruits). Consumption of food by any of the household member from any of 12 food groups in the last 24 h was also assessed and the household DDS was determined. The food groups were cereals; roots and tubers; vegetables; fruits; meat, poultry, offal; eggs; fish and seafood; pulses/legumes/nuts; milk and milk products; oils/ fats; sugar/honey and miscellaneous [23].

### Other covariates

Data including socio-demographic (such as maternal age, maternal education, sex of the head of the household and paternal education), economic (household asset), environmental factors (such as water source and latrine type), health service utilization (such as place of delivery for index child), and household characteristics (such as the number of under-five children living in a household) were collected. Different socio-economic indicators were combined using principal component analysis to construct household wealth. The factor scores were divided into quantiles (poorest, poorer, middle, richer and richest) to indicate the relative socio-economic status of the participants. The highest level of education achieved was categorized into no education, primary, and secondary and above. Water source was classified as piped, other improved and unimproved. The type of functional latrine used in the household was categorized into traditional pit latrine, improved latrine and no facility/bush/field.

### Anthropometry and dietary data analyses

Height-for-age z score (HAZ), an indicator of linear growth, was compared with reference data from the World Health Organization (WHO) Multicentre Growth Reference Study Group, 2006 [24] using the ENA (Emergency Nutrition Assessment) SMART (Standardized Monitoring and Assessment of Relief and Transitions) 2011 software. Children whose HAZ is <−2 SD from the median of the WHO reference population were considered stunted (short for their age). In our analysis, both HAZ (continuous) and stunting (categorical; stunted = 1/not stunted = 0) were used as outcome variables. Maternal body mass index (BMI) was calculated based on the measured weight (kg) and height (meters) (weight [kg]/(height[meters])^2^).

For children aged 6 to 23, and 24 to 59 months, the minimum acceptable DDS was defined as consuming food from four or more of the standardized set of seven (6 to 23 months) or nine (24 to 59 months) food groups on the preceding day of the survey [7, 25]. For women (mothers/caregivers of the index children), a threshold of at least 5 food groups of the 10 was considered acceptable [8]. The scores of household DDS were continuous, ranging from 0 to 12, based on whether any of the members of the household consumed any of the 12 food groups. Minimum household DDS was not determined because a dichotomous indicator has not been developed [8, 23]. However, we assumed that consumption of food groups above the median number as adequate. Nine Household Food Insecurity Access Scale (HFIAS) generic questions were used with a dichotomized response (0 = no/1 = yes) to assess food insecurity [9]. Each of the questions were asked with a recall period of four weeks (30 days). If a respondent answers “yes” to any of the above nine questions, frequency-of-occurrence questions were asked to determine whether the condition happened rarely (1 = once or twice), sometimes (2 = three to ten times) or often (3 = more than ten times). The insecurity status was categorized into four groups (secured, mild, moderate, and severe) using the Food and Nutrition Technical Assistance (FANTA) algorithm [9].

Dietary patterns were identified by *polychoric (tetrachoric)* analysis—a family of factor analysis which uses a *tetrachoric* correlation matrix to construct latent variables from dichotomized (binary) observed data [26]. For each of the dietary patterns, factor scores were assigned for all study participants. Factor scores show the relative position of the study participants in each of the identified patterns, thus reflecting adherence to the patterns. Pattern-specific factor scores are calculated as the sum of the products of the factor loading coefficients and standardized daily consumption of food and nutrient groups related to the pattern. The factor scores were orthogonally (varimax) rotated to create less correlation among the patterns and to facilitate their interpretability. Participants were then assigned into tertiles (first [lowest adherence]; second; and third [highest adherence] tertiles) based on their factor scores. Eigenvalues (>1), scree plots, and interpretability of the factors were used to determine the number of dietary and nutrient patterns. Factor loadings (the correlation between each pattern and the food and nutrient groups) were calculated. Percentages of variances (the variations that were explained by the identified dietary and nutrient patterns) were also computed.

### Statistical analyses

The chi-square (categorical variables), ANOVA (normally distributed continuous variables) and Kruskal-Wallis (continuous but not normally distributed) tests were used to compare differences of proportions, means and medians, respectively, between groups. Principal component analysis (PCA) was used to compute economic status (in quintiles) of households.

To assess the associations of household, maternal and child dietary diversity and patterns with HAZ and childhood stunting, β coefficients and the prevalence ratio (PR) with their corresponding 95% confidence intervals (CIs) were determined using multilevel linear and Poisson regression models, respectively [27]. Since the data were collected using a multi-stage cluster sampling technique, stunting could potentially be correlated in clusters (EAs). We, therefore, used a two-level model with individual factors as level 1 and geographical areas (EAs) at level 2 (random effects). A stepwise backward elimination of covariates in the models was conducted and potential factors were retained at *p-value* < 0.20. This method was used for both individual and community level factors. Dietary diversity and pattern scores were treated as categorical (model 1) and continuous (model 2) variables. Estimates of associations were adjusted for socio-demographic factors (child age, sex, maternal age and education, number of under-five children in a household), maternal anthropometry (height and BMI), infant and young child feeding practices (exclusive breastfeeding) and household food security at level 1. At level 2, water source was included. Model fit was assessed using Akaike’s (AIC) and Bayesian (BIC) information criteria. We tested interactions between DDSs, dietary patterns, other covariates with HAZ and stunting using multiplicative terms. We conducted sensitivity analysis: 1) by labelling missing values of covariates as “missing” and including in the models; 2) by including and excluding covariates (such as household wealth, paternal education, place of delivery and latrine type). Further, the association between joint classifications of tertiles of dietary patterns and HAZ was explored. Statistical analyses were performed using Stata version 14.1 (Stata Corporation, College Station, TX, USA). A 2-sided *t-test* value of *P < 0.05* was considered statistically significant.

## Results

### Participant characteristics

Almost half of the children (1805, 47.7%) were female. Only 3.1% of the households had a female head. In 2353 (90.8%) of households, there was only one child aged under five. The median maternal age was 29.0 years (IQR = 25.0, 35.0). More than half (2069; 54.6%) of the mothers were illiterate. The mean maternal BMI was 20.2 kg/m^2^ (SD = 2.4). Almost two-thirds (2371; 62.6%) of mothers delivered the index child at home. The prevalence of stunting among children aged 0–59 months was 38.5% with a mean (SD) HAZ of 1.6 (1.8). A fifth (777; 20.5%) of the study participants had missing values of household income (Table 2).

**Table 2:** Characteristics of study participants by age of children in Ethiopia, 2014

Characteristics	Overall	0–5 months	6–23 months	24–59 months	*p*-value
N	3788	601	896	2287	
Sex of children					
Male	1850 (48.8%)	284 (47.3%)	438 (48.9%)	1126 (49.2%)	0.680
Female	1805 (47.7%)	294 (48.9%)	433 (48.3%)	1076 (47.0%)	
Missing	133 (3.5%)	23 (3.8%)	25 (2.8%)	85 (3.7%)	
Sex of household head					
Male	3438 (90.8%)	560 (93.2%)	820 (91.5%)	2055 (89.9%)	0.098
Female	231 (6.1%)	26 (4.3%)	53 (5.9%)	151 (6.6%)	
Missing	119 (3.1%)	15 (2.5%)	23 (2.6%)	81 (3.5%)	
Number of under 5 children in the household					
One Child	2353 (62.1%)	259 (43.1%)	505 (56.4%)	1585 (69.3%)	<0.001
Two children	1313 (34.7%)	300 (49.9%)	372 (41.5%)	641 (28.0%)	
More than three children	97 (2.6%)	40 (6.7%)	18 (2.0%)	39 (1.7%)	
Missing	25 (0.7%)	2 (0.3%)	1 (0.1%)	22 (1.0%)	
Maternal age, median (IQR)	29.0 (25.0, 35.0)	27.0 (23.0, 32.0)	28.0 (23.0, 33.0)	30.0 (25.0, 35.0)	<0.001
Maternal education					
No education	2069 (54.6%)	274 (45.6%)	454 (50.7%)	1339 (58.5%)	<0.001
Primary	1364 (36.0%)	265 (44.1%)	351 (39.2%)	746 (32.6%)	
Secondary and above	193 (5.1%)	41 (6.8%)	50 (5.6%)	102 (4.5%)	
Missing	162 (4.3%)	21 (3.5%)	41 (4.6%)	100 (4.4%)	
Paternal education					
No education	1283 (33.9%)	177 (29.5%)	282 (31.5%)	823 (36.0%)	0.004
Primary	1644 (43.4%)	293 (48.8%)	397 (44.3%)	952 (41.6%)	
Secondary and above	417 (11.0%)	73 (12.1%)	106 (11.8%)	238 (10.4%)	
Missing	444 (11.7%)	58 (9.7%)	111 (12.4%)	274 (12.0%)	
Maternal body-mas index (kg/m^2^), mean (SD)	20.2 (2.4)	20.9 (2.4)	20.0 (2.3)	20.1 (2.4)	<0.001
Maternal Height (meter), mean (SD)	1.6 (0.1)	1.6 (0.1)	1.6 (0.1)	1.6 (0.1)	0.550
Place of delivery					
Home	2371 (62.6%)	263 (43.8%)	483 (53.9%)	1623 (71.0%)	<0.001
Health facility	1346 (35.5%)	327 (54.4%)	398 (44.4%)	619 (27.1%)	
Other	71 (1.9%)	11 (1.8%)	15 (1.7%)	45 (2.0%)	
Water source					
Piped water	1694 (44.7%)	279 (46.4%)	399 (44.5%)	1014 (44.3%)	0.740
Other improved	1104 (29.1%)	168 (28.0%)	253 (28.2%)	683 (29.9%)	
Unimproved	990 (26.1%)	154 (25.6%)	244 (27.2%)	590 (25.8%)	
Latrine type					
Traditional pit latrine	3087 (81.5%)	493 (82.0%)	735 (82.0%)	1857 (81.2%)	0.015
Improved latrine	25 (0.7%)	10 (1.7%)	3 (0.3%)	12 (0.5%)	
No facility/bush/field	676 (17.8%)	98 (16.3%)	158 (17.6%)	418 (18.3%)	
Income quantile					
Poorest	603 (15.9%)	87 (14.5%)	151 (16.9%)	364 (15.9%)	0.470
Poorer	602 (15.9%)	96 (16.0%)	134 (15.0%)	372 (16.3%)	
Middle	602 (15.9%)	84 (14.0%)	142 (15.8%)	376 (16.4%)	
Richer	602 (15.9%)	109 (18.1%)	136 (15.2%)	356 (15.6%)	
Richest	602 (15.9%)	106 (17.6%)	144 (16.1%)	351 (15.3%)	
Missing	777 (20.5%)	119 (19.8%)	189 (21.1%)	468 (20.5%)	
Stunted					
No	2161 (57.0%)	510 (84.9%)	539 (60.2%)	1109 (48.5%)	<0.001
Yes	1459 (38.5%)	45 (7.5%)	330 (36.8%)	1084 (47.4%)	
Missing	168 (4.4%)	46 (7.7%)	27 (3.0%)	94 (4.1%)	
Height-for-age z-score, mean (SD)	−1.6 (1.8)	0.0 (1.6)	−1.5 (1.6)	−2.0 (1.6)	<0.001
Underweight					
No	2986 (78.8%)	563 (93.7%)	704 (78.6%)	1715 (75.0%)	<0.001
Yes	802 (21.2%)	38 (6.3%)	192 (21.4%)	572 (25.0%)	
Weight-for-age z-score, mean (SD)	−1.0 (2.3)	0.2 (3.7)	−1.0 (2.3)	−1.3 (1.6)	<0.001

### Dietary patterns

**Fig. 2:**
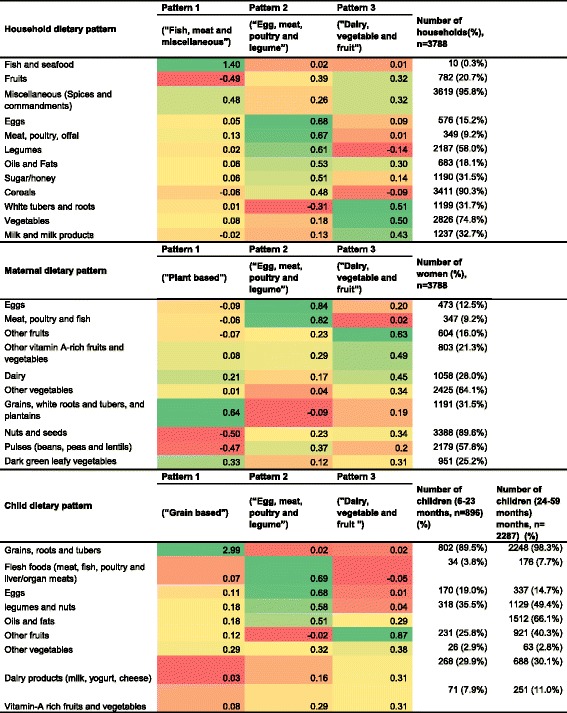
Household, maternal and child dietary patterns and corresponding factor loadings and proportion of food groups. The colour gradation reflects how large and in which direction was the correlation between the food groups and the dietary patterns. Deep green colour refers a relatively higher correlation (a higher intake) of the food groups with the corresponding patterns. Deep red refers relatively a lower correlation (a lower intake) of the food groups with the corresponding dietary patterns

Figure 2 depicts household, maternal and child dietary patterns and corresponding factor loadings. For each, we identified three dietary patterns. Pattern 2 (“egg, meat, poultry and legume”) and pattern 3 (“dairy, vegetable and fruit pattern”) were similar for all groups. The “egg, meat, poultry and legume pattern” was characterized by a high intake of eggs, meats, legumes, cereals, oils, fats and sweets. The “dairy, vegetable and fruit based” pattern was characterized by a high intake of fruits tubers, roots, vegetables and milk and milk products. A “plant-based pattern” and a “dairy, vegetable and fruit pattern” were identified for mothers. While these two patterns appeared to be similar, the “plant-based” pattern was however characterized by a high intake of grains, tubers and leafy vegetables. Individual food items used for food groupings and the proportion of food groups consumed by households, mothers and children are depicted in Additional file 1: Tables S1, S2 and S3.

### DDSs, dietary patterns, household food security and stunting

Table 3 and Additional file 1: Table S4 show the DDSs of households, mothers and children by whether or not stunting was present. Three-quarters (2859; 75.5%) of the mothers had a DDS less than five. Only 13.1% of children aged 6–23 months had a DDS greater than or equal to four. The proportion of children aged 24–59 months who had a DDS less than or equal to three was 61.7% (1411). The proportion of food secure households was 46.0% (1744). A marginally significant statistical difference in the proportion of stunting was found between those children with a household DDS < =5 and >5 (*p* = 0.045). The prevalence of stunting was significantly different by maternal DDS (*p* = 0.005). No significant difference in the prevalence of stunting was found between the DDSs of children aged 6–23 and 24–59 months.

**Table 3:** Household, maternal and child dietary diversity scores, food security and breastfeeding by stunting status in Ethiopia, 2014

Characteristics	Total	Normal	Stunted	*p*-value
N	3788	2161	1459	
Household dietary diversity score (HDDS), median (IQR)	5.0 (4.0, 7.0)	5.0 (4.0, 7.0)	5.0 (4.0, 6.0)	0.004
HDDS category				
< =5 HDDS	2031 (53.6%)	1131 (52.3%)	813 (55.7%)	0.045
> 5 HDDS	1757 (46.4%)	1030 (47.7%)	646 (44.3%)	
Women (DDS-W), median (IQR)	3.0 (2.0, 4.0)	3.0 (2.0, 5.0)	3.0 (2.0, 4.0)	<0.001
DDS-W category				
< 5 DDSW	2859 (75.5%)	1595 (73.8%)	1137 (77.9%)	0.005
> =5 DDSW	929 (24.5%)	566 (26.2%)	322 (22.1%)	
DDS 6–23 months, median (IQR)	2.0 (1.0, 3.0)	2.0 (0.0, 3.0)	2.0 (2.0, 3.0)	<0.001
DDS 6–23 months category				
0–3 food groups	779 (86.9%)	474 (87.9%)	282 (85.5%)	0.552
4–7 food groups	117 (13.1%)	65 (12.1%)	48 (14.6%)	
Child DDS (24–59 months), median (IQR)	3.0 (1.0, 4.0)	2.0 (0.0, 4.0)	3.0 (2.0, 4.0)	<0.001
Child DDS (24–59 months) category				
0–3 food groups	1411 (61.7%)	661 (59.6%)	684 (63.1%)	0.054
4–9 food groups	876 (38.3%)	448 (40.4%)	400 (36.9%)	
Household food security				
Food Secure	1744 (46.0%)	1026 (47.5%)	621 (42.6%)	0.005
Mildly Food Insecure Access	344 (9.1%)	201 (9.3%)	133 (9.1%)	
Moderately Food Insecure Access	1103 (29.1%)	626 (29.0%)	438 (30.0%)	
Severely Food Insecure Access	574 (15.2%)	298 (13.8%)	256 (17.5%)	
Missing	23 (0.6%)	10 (0.5%)	11 (0.8%)	
Exclusive breast feeding	2719 (71.8%)	1592 (73.7%)	998 (68.4%)	0.001

*HDDS* household dietary diversity score, *DDSW* women dietary diversity score

Except for the “fish, meat and miscellaneous” dietary pattern (*p* = 0.097), there were significant differences in the prevalence of stunting across the tertiles of the other household dietary patterns. The prevalence of stunting (41.6%) in the first tertile of household “dairy, vegetable and fruit” dietary pattern was higher compared to the third tertile (33.3%) (*p* < 0.001). Children in the third tertile of maternal and child “dairy, vegetable and fruit” dietary pattern were less likely to be stunted compared to the first and second tertiles (*p* = 0.002) (Table 4).

**Table 4:** Stunting prevalence among children across tertiles of household, maternal and child nutrient pattern scores in Ethiopia, 2014

	Tertiles of dietary patterns			
	T1	T2	T3	
Household dietary patterns				
	Pattern 1 (“fish, meat and miscellaneous”)			
n	1298	1259	1154	
Normal	715 (55.1%)	734 (58.3%)	668 (57.9%)	0.097
Stunted	533 (41.1%)	472 (37.5%)	424 (36.7%)	
Missing	50 (3.9%)	53 (4.2%)	62 (5.4%)	
	Pattern 2 (“egg, meat, poultry and legume”)			
n	1327	1291	1093	
Normal	760 (57.3%)	762 (59.0%)	595 (54.4%)	0.044
Stunted	516 (38.9%)	464 (35.9%)	449 (41.1%)	
Missing	51 (3.8%)	65 (5.0%)	49 (4.5%)	
	Pattern 3 (“dairy, vegetable and fruit”)			
n	1441	1052	1218	
Normal	776 (53.9%)	584 (55.5%)	757 (62.2%)	<0.001
Stunted	599 (41.6%)	425 (40.4%)	405 (33.3%)	
Missing	66 (4.6%)	43 (4.1%)	56 (4.6%)	
Maternal dietary patterns				
	Pattern 1 (“plant-based”)			
n	1270	1232	1235	
Normal	683 (53.8%)	701 (56.9%)	749 (60.6%)	0.002
Stunted	529 (41.7%)	481 (39.0%)	431 (34.9%)	
Missing	58 (4.6%)	50 (4.1%)	55 (4.5%)	
	Pattern 2 (“egg, meat, poultry and legume”)			
n	1264	1291	1182	
Normal	755 (59.7%)	714 (55.3%)	664 (56.2%)	0.020
Stunted	447 (35.4%)	528 (40.9%)	466 (39.4%)	
Missing	62 (4.9%)	49 (3.8%)	52 (4.4%)	
	Pattern 3 (“dairy, vegetable and fruit”)			
n	1262	1233	1242	
Normal	679 (53.8%)	708 (57.4%)	746 (60.1%)	0.002
Stunted	530 (42.0%)	476 (38.6%)	435 (35.0%)	
Missing	53 (4.2%)	49 (4.0%)	61 (4.9%)	
Child dietary patterns (6–59 months of age)				
	Pattern 1 (“grain-based”)			
	1274	950	963	
Normal	649 (50.9%)	509 (53.6%)	493 (51.2%)	0.430
Stunted	584 (45.8%)	409 (43.1%)	421 (43.7%)	
Missing	41 (3.2%)	32 (3.4%)	49 (5.1%)	
	Pattern 2 (“egg, meat, poultry and legume”)			
n	1255	999	933	
Normal	640 (51.0%)	538 (53.9%)	473 (50.7%)	0.249
Stunted	561 (44.7%)	422 (42.2%)	431 (46.2%)	
Missing	54 (4.3%)	39 (3.9%)	29 (3.1%)	
	Pattern 3 (“dairy, vegetable and fruit”)			
n	1136	1029	1022	
Normal	577 (50.8%)	499 (48.5%)	575 (56.3%)	0.002
Stunted	505 (44.5%)	494 (48.0%)	415 (40.6%)	
Missing	54 (4.8%)	36 (3.5%)	32 (3.1%)	

### Associations of DDSs and dietary patterns with HAZ and stunting

After adjusting for potential individual and community level factors, no significant associations between household, maternal and child DDSs with stunting (HAZ) was found. Children in the third tertile of the household “dairy, vegetable and fruit” pattern had a 0.16 (β = 0.16; 95% CI: 0.02, 0.30) increase in HAZ compared to those in the first tertile. Similarly, the prevalence of stunting among children in the third tertile of the pattern was lower (PR = 0.83; 95% CI: 0.72–0.95) compared to those in the first tertile. Those children in the second (β = −0.17; 95% CI: -0.31, −0.04) and third (β = −0.16; 95% CI: -0.30, −0.02) tertiles of maternal “egg, meat, poultry and legume” pattern had a significantly lower HAZ compared to those in the first tertile (Table 5 and Additional file 1: Table S5).

**Table 5:** Adjusted β coefficients (95% confidence interval) for the associations of household, maternal and child dietary diversity scores and tertiles of dietary pattern scores with childhood height-for-age z score in Ethiopia, 2014

	Adjusted β coefficient (95% confidence interval) for height-for-age z score				
Household					
Dietary diversity score	<=4	> = 5		*P* value	AIC (BIC)
	Reference	0.03 (−0.80, 0.14)		0.624	12,722 (12850)
	Tertiles				
Dietary patterns	T1	T2	T3	P for trend	AIC (BIC)
Pattern 1 (“fish, meat and miscellaneous”)	Reference	−0.01 (−0.15, 0.12)	−0.07 (−0.21, 0.07)	0.312	12,466 (12600)
Pattern 2 (“egg, meat, poultry and legume”)	Reference	−0.001 (−0.14, 0.13)	−0.03 (−0.19, 0.12)	0.681	12,467 (12601)
Pattern 3 (“dairy, vegetable and fruit”)	Reference	0.07 (−0.07, 0.20)	0.16 (0.02, 0.30)*	0.026	12,462 (12597)
Maternal					
Dietary diversity score	<=4	> = 5		P value	AIC (BIC)
	Reference	0.03 (−0.10, 0.16)		0.672	12,722 (12851)
	Tertiles				
Dietary patterns	T1	T2	T3	P for trend	AIC (BIC)
Pattern 1 (“plant-based”)	Reference	0.07 (−0.07, 0.20)	0.09 (−0.06, 0.23)	0.229	12,564 (12699)
Pattern 2 (“egg, meat, poultry and legume”)	Reference	−0.17 (−0.31, −0.04)*	−0.16 (−0.30, −0.02)*	0.025	12,558 (12693)
Pattern 3 (“dairy, vegetable and fruit”)	Reference	0.06 (−0.07, 0.20)	0.10 (−0.05, 0.25)	0.229	12,564 (12698)
Children aged 6–23 months					
Dietary diversity score	<=3	> = 4		P value	AIC (BIC)
	Reference	−0.26 (−0.61, 0.78)		0.130	3107 (3196)
	Tertiles				
Dietary patterns	T1	T2	T3	P for trend	AIC (BIC)
Pattern 1 (“grain-based”)	Reference	0.07 (−0.18, 0.32)	−0.20 (−0.50, 0.10)	0.290	3124 (3219)
Pattern 2 (“egg, meat, poultry and legume”)	Reference	0.03 (−0.30 (0.32)	−0.02 (−0.27, 0.24)	0.892	3127 (3222)
Pattern 3 (“dairy, vegetable and fruit”)	Reference	−0.001 (−0.25, 0.25)	0.02 (−0.27, 0.32)	0.890	3128 (3222)
Children aged 24–59 months					
Dietary diversity score	<=3	> = 4		P value	AIC (BIC)
	Reference	0.12 (−0.02, 0.27)		0.095	7641 (7748)
	Tertiles				
Dietary patterns	T1	T2	T3	P for trend	AIC (BIC)
Pattern 1 (“grain-based”)	Reference	0.08 (−0.09, 0.25)	0.04 (−0.14, 0.21)	0.685	7663 (7776)
Pattern 2 (“egg, meat, poultry and legume”)	Reference	0.13 (−0.3, 0.30)	−0.05 (−0.23, 0.13)	0.668	7659 (7772)
Pattern 3 (“dairy, vegetable and fruit”)	Reference	−0.03 (−0.20, 0.14)	0.22 (0.06, 0.39)**	0.007	7654 (7766)
Children aged 6–59 months					
	Tetiles				
Dietary patterns	T1	T2	T3	P for trend	AIC (BIC)
Pattern 1 (“grain-based”)	Reference	0.10 (−0.04, 0.25)	−0.04 (−0.19, 0.11)	0.643	10,746 (10871)
Pattern 2 (“egg, meat, poultry and legume”)	Reference	−0.07 (−0.7, 0.21)	−0.02 (−0.17, 0.13)	0.841	10,748 (10874)
Pattern 3 (“dairy, vegetable and fruit”)	Reference	−0.02 (−0.16, 0.13)	0.19 (0.04, 0.33)*	0.014	10,741 (10867)

The β coefficients were adjusted for both individual (maternal body mass index, age (if applicable), household food security, number of under-five children in a household, maternal education, maternal height, exclusive breast feeding) and community-level patterns (water source)

*P* for trend was determined by including the tertiles of dietary patterns as continuous variables

*AIC* Akaike’s information criterion, *BIC* Bayesian information criterion

*p < 0.05; **p < 0.01

**Fig. 3:**
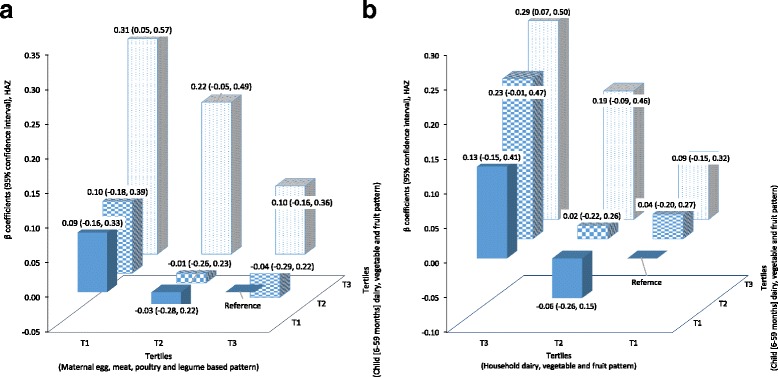
Multivariable adjusted β coefficient and 95% confidence interval of fractures in joint classified participants across nine strata formed with the tertiles of dietary patterns. The β coefficients were adjusted for both individual (maternal body mass index, age (if applicable), household food security, number of under-five children in a household, maternal education, maternal height, exclusive breast feeding) and community-level patterns (water source)

A 0.22 (β = 0.22; 95% CI: 0.06, 0.39) and 0.19 (β = 0.19; 95% CI: 0.04, 0.33) increase in HAZ was found for those in the third tertiles of the “dairy, vegetable and fruit” patterns for children 24–59 months and 6–59 months, respectively. The AIC and BIC were significantly lower for the household and maternal dietary patterns compared to the corresponding DDSs (Table 5). In the joint classification, children in the first tertile of maternal “egg, meat, poultry and legume” and the third tertile of child “dairy, vegetable and fruit” patterns had a 0.31 (β = 0.31; 95% CI: 0.05, 0.57) increase in HAZ compared to the respective third and first tertiles of the patterns. A 0.29 (β = 0.29; 95% CI: 0.07, 0.50) increase in HAZ was found for those children in the third tertiles of both the maternal and child “dairy, vegetable and fruit” dietary patterns, compared to the first tertiles of the patterns (Fig. 3).

The sensitivity analysis, which was undertaken by labelling missing values as “missing” and including them in the analysis, as well as incorporating additional covariates, did not change the findings materially. There were no significant interactions between the DDSs and dietary patterns, and the other covariates and HAZ (stunting). The interactions among dietary patterns for each of the levels (household, maternal and children) were also not significant (data not shown).

## Discussion

For households, mothers and children, we computed the DDSs and identified three dietary patterns for each. At all levels, a dietary pattern characterized by high intake of dairy, vegetables and fruits was positively associated with HAZ and inversely related to stunting. In addition, a maternal “plant-based” pattern (characterized by high intake of grains, white tubers, roots, plantains and dark leafy vegetables) was inversely and significantly associated with childhood stunting. Statistically non-significant associations were found between household, maternal and child DDSs and stunting.

Dietary patterns show a better picture of eating habits compared to DDSs by reflecting mainly the behavioural aspect of food consumption that has a synergistic effect on health. Evidence shows that dietary patterns identified by factor analysis are associated with several non-communicable diseases [28, 29], highlighting the plausibility and validity of the approach. In low- and middle-income countries, a study also indicated that the application of factor analysis, using disaggregated food expenditure data to explore food consumption patterns, is an important approach to identify patterns and food groups that predict children’s nutritional status [19]. The dietary patterns are defined based on the factor loadings of individual food items which contribute at different levels. Unlike DDSs, factor analysis is an a posteriori statistical analysis method that creates unrelated food patterns that could potentially be associated with an outcome [17]. In addition, using this approach, it is possible to assess the relative intake level of the individual food groups within a dietary pattern. Dietary patterns defined the overall characteristics of the dietary habits of the study groups. In this study, we found that the “dairy, vegetable and fruit” pattern was a common feature of household, maternal and childhood dietary habits.

The results indicate that a pattern characterised by a high intake of dairy, vegetables and fruits was positively associated with HAZ (inversely related to stunting). However, the proportion of households (32.7%), mothers (28.0%) and children (30.0%) consuming dairy products was low. In a recent study in Malawi, it was reported that frequent milk intake during pregnancy was positively associated with birth size [30]. In Ethiopia, a higher intake of cow’s milk, in addition to cereals and/or legumes, was associated with a higher length-for-age z-scores among children aged 5–11 months [31]. In our study area, cow’s milk is the most commonly consumed type of dairy product. It is believed that the milk contains important nutrients, including protein, calcium and vitamin A, which are important for development and bone growth [32]. A systematic review of dairy consumption and physical growth has shown that a daily intake of 245 ml of milk is associated with 0.4 cm increase in height per annum compared to non-consumers [33]. It was also reported that low consumption of vegetables and fruits was associated with stunting and poor linear growth in children aged 6–23 months [34]. This implies that, in addition to available interventions to increase dietary diversity, targeting to increase accessibility and consumption of dairy, vegetables and fruits specifically, could have an important contribution to the reduction of stunting prevalence.

Unlike other studies [14, 15, 35], we found a non-significant association of household, maternal and child DDSs with stunting. This difference could be explained by the differences in sample size, sampling and analysis methods. Particularly, with regard to the analysis methods, we used a multilevel Poisson regression model to determine the level of associations. This method allows for controlling for the geographical clustering effect of the samples, and accounting for potential correlations, under the premise that variations in childhood stunting could be due to both individual and community level factors [36]. Most studies that found a positive association between DDS and stunting did not consider this clustering effect [16, 37]. The association may also be confounded by income [38, 39]. However, in this study, even when we adjusted for income, the association still remained statistically non-significant.

A study by *Daniels* et al. suggested that the addition of portion size as part of data collection could improve the correlation of DDSs with nutrient adequacy [40]. Another study among Zambian infants suggested that although dietary diversity had a positive effect on linear growth, micronutrient adequacy among those who consumed fortified foods may be more accurately assessed using other food indicators [41]. In Eastern Kenya, a study reported that child DDS was not associated with childhood stunting [42]. In Ethiopia, while household food group expenditure index (identified using factor analysis) significantly predicted HAZ (β = 0.067; *p* = 0.03), dietary diversity was only marginally associated with HAZ (β = 0.037; *p* = 0.05) [19]. DDSs are important indicators of dietary quality in terms of micronutrient density and adequacy. However, DDSs only measure one dimension of dietary quality. Macronutrients (for instance, protein) also have an important role in growth and development in children [8]. Although DDS is an important approach to measure dietary quality, we recommend that the use of a posteriori dietary data analysis methods (such as factor analysis) as an alternative or complementary method, can give a further insight into the eating behaviours of a population group. These approaches are also important to understand the relative contribution of foods in a pattern that have a potential link with disease outcomes or nutritional status, eventually leading to identifying specific food items, which are most important in determining an outcome of interest (a disease or nutritional status).

Measures taken to ensure the quality of the data are a major strength of the study. Before, during and after the data collection, all possible quality control measures, including intensive training of data collectors, use of standard procedures and tools, intensive and supportive supervision and standardization of anthropometric measurements to minimize bias and associated errors were implemented. The use of qualitative dietary data without portion size and limited food items for the identification of dietary patterns in the factor analysis could be a limitation. Therefore, further validation studies are needed. In addition, due to the cross-sectional design, we cannot claim a cause-effect relationship between dietary patterns and stunting.

## Conclusions

Identification of dietary patterns using a posteriori dietary analysis methods can be an alternative and feasible method of diet quality assessment in LICs as an alternative approach to DDSs. We found that, while DDSs are not significantly associated with HAZ (stunting), a dietary pattern characterized by a high intake of dairy, vegetables and fruits by households, mothers and children is positively associated with HAZ and inversely associated with stunting. These findings could be of importance in developing food-based interventions targeting households, mothers and children. In addition, the study suggests an alternative approach of analysing dietary data to determine dietary quality using an a posteriori method with the same data collected for DDSs. More research is warranted to confirm the findings.

## Additional file

Additional file 1: **Table S1.** Components of food groups used for dietary pattern (household and maternal), 2014. **Table S2.** Components of food groups used for dietary pattern (children), 2014. **Table S3.** Proportion children, mothers and household by dietary diversity scores (DDS), 2014. **Table S4.** Dietary diversity and stunting in Ethiopia, 2014. **Table S5.** Prevalence ratios (95% confidence interval) for the associations of household, maternal and child dietary diversity scores and dietary patterns with childhood stunting in Ethiopia, 2014 (PDF 173 kb)

## Abbreviations

AIC: Akaike’s information criterion
BIC: Bayesian information criterion
BMI: Body mass index
*CI*: Confidence interval
DDS: Dietary diversity score
EA: Enumeration area
FANTA: Food and Nutrition Technical Assistance
HAZ: Height-for-age z score
LIC: Low income countries
PPS: Probability proportional to size
PR: Prevalence ratio
SNNP: South Nation and Nationalities and People
UNICEF: United Nations International Children’s Emergency Fund
WHO: World Health Organization
