# Supplementary material for: Associations of childhood, maternal and household dietary patterns with childhood stunting in Ethiopia: proposing an alternative and plausible dietary analysis method to dietary diversity scores
Source: Nutr J. 2018 Jan 29;17:14. doi: 10.1186/s12937-018-0316-3 (PMC5789646; doi:10.1186/s12937-018-0316-3)
Supplement: Additional file 1: — Table S1. Components of food groups used for dietary pattern (household and maternal), 2014. Table S2. Components of food groups used for dietary pattern (children), 2014. Table S3. Proportion children, mothers and household by dietary diversity scores (DDS), 2014. Table S4. Dietary diversity and stunting in Ethiopia, 2014. Table S5. Prevalence ratios (95% confidence interval) for the associations of household, maternal and child dietary diversity scores and dietary patterns with childhood stunting in Ethiopia, 2014 (PDF 173 kb) [file 12937_2018_316_MOESM1_ESM.pdf]

Supplementary Table 1: Components of food groups used for dietary pattern (household and maternal), 2014

Food group	Components
cereals	Rice, bread made of wheat, puffed rice, injera, pressed rice, noodles, or any other foods made with rice, wheat, maize/corn, or other locally available grains
vitamin a rich vegetables and tubers	Pumpkin, carrots, sweet potatoes that are orange and yellow inside
white tubers and roots or other starchy foods	Potatoes, white yams, white sweet potato (not orange inside), potato crisps or other foods made from roots (not orange or yellow roots)
dark green leafy vegetables	Dark green leafy vegetables, including spinach, kale, costa,
other vegetables	Other vegetables (e.g., squash, eggplant, green papaya, cauliflower, cabbage, onion, (beans)
vitamin a rich fruits	Ripe mangoes, ripe papaya
other fruits	banana, apples, guava, oranges, other citrus fruits, pineapple, watermelon, olives, grapes, (grapefruit) berries, , plum
beef, goat, lamb, chicken, duck or other birds, liver, kidney, heart, or other organ meats	
eggs	Eggs of different birds – chicken, duck, turkey etc.; with yolk, without yolk
fish	Big/small fresh or dried fish or shellfish
any foods made from beans, peas, or lentils	Beans, peas, lentils, other pulses, soybeans, peas
milk and milk products	Milk, cheese, yogurt or other milk products
oils and fats	Oil, fats or butter added to food or used for cooking including ghee
sweets	Sugar, molasses, honey, misti, cold drinks, chocolates, candies, biscuits
spices, condiments, beverages	Spices (cumin, coriander, salt), condiments (pickles, chutney), coffee, tea, etc.

Supplementary Table 2: Components of food groups used for dietary pattern (children), 2014

No	Food items
1	Any porridge
2	Any gruel
3	Any commercially fortified food (Cerifam, Fafa, Farmixt milk, Favena, Berta, Mother's Choice)
4	Bread, pasta, rice, noodles, biscuits, cookies, or any other foods made from oats, maze, barley, wheat, sorghum, millet or other grain
5	Injera or kita
6	Any white potatoes, white yams, Bulla, Kocho, Kasava or any other food made from roots
7	Any pumpkin, carrot, squash or sweet potato that are yellow or orange inside
8	Dark green leafy vegetables (example: Kale, spinach or Amaranth leaves)
9	Any other vegetables (starchy vegetables: plantain)
10	Any liver, kidney, heart or organ meats
11	Any meat (which does not include any organ meats, dry meat, any chicken ducks or other birds)
12	Any dry meat
13	Any chicken ducks or other birds
14	Any eggs
15	Any fresh or dried fish or shell fish
16	Any food made from beans, peas, lentil or pulses
17	Any nuts or seeds such as peanuts, sesame, sunflower seeds
18	Any milk product like cheese, yogurt
19	Any food made from oil, fat or butter
20	Any Ready to use therapeutic foods (like plumpy nut, F100)
21	Candies or chocolates, cakes or donuts
22	Any other solid or semi-solid food
23	Any iron-containing tablet, syrup or sprinkles
24	Kolo/Chips/Crisps
25	Biscuits/Cookies
26	Popcorn
27	Juice/Soda/fizzy drink

Supplementary Table 3: Proportion children, mothers and household by dietary diversity scores (DDS), 2014

Dietary diversity scores	Household DDS	DDS-women	DDS (6-23 months)	DDS (24-59 months)
	3788			
0	8 (0.2%)	32 (0.8%)	87 (2.7%)	84 (2.6%)
1	19 (0.5%)	253 (6.7%)	604 (19.0%)	429 (13.5%)
2	127 (3.4%)	792 (20.9%)	1200 (37.7%)	616 (19.3%)
3	364 (9.6%)	1006 (26.6%)	774 (24.3%)	920 (28.9%)
4	623 (16.4%)	776 (20.5%)	327 (10.3%)	647 (20.3%)
5	890 (23.5%)	455 (12.0%)	142 (4.5%)	292 (9.2%)
6	805 (21.3%)	257 (6.8%)	45 (1.4%)	140 (4.4%)
7	490 (12.9%)	120 (3.2%)	8 (0.3%)	47 (1.5%)
8	290 (7.7%)	72 (1.9%)		8 (0.3%)
9	128 (3.4%)	23 (0.6%)		4 (0.1%)
10	40 (1.1%)	2 (0.1%)		
11	4 (0.1%)			
12	0 (0.0%)			

Supplementary Table 4: Dietary diversity and stunting in Ethiopia, 2014

**HHDDS (household DDS)**		**<=5**	**>5**	**P-value**
N		2031	1757	
Stunting	Normal	1131 (55.7%)	1030 (58.6%)	0.045
	Stunted	813 (40.0%)	646 (36.8%)	
	Missing	87 (4.3%)	81 (4.6%)	

**Maternal DDS**	**Level**	**<5 ddsw**	**>=5 ddsw**	**p-value**
N		2859	929	
stunting	Normal	1595 (55.8%)	566 (60.9%)	0.005
	Stunted	1137 (39.8%)	322 (34.7%)	
	Missing	127 (4.4%)	41 (4.4%)	

**Child (6-23 months) DDS**	**Level**	**0-3 food groups**	**4-7 food groups**	**p-value**
N		779	117	
stunting	Normal	474 (60.8%)	65 (55.6%)	0.29
	Stunted	282 (36.2%)	48 (41.0%)	
	Missing	23 (3.0%)	4 (3.4%)	

**Child (24-59 months) DDS**	**Level**	**0-3 food groups**	**4-9 food groups**	**p-value**
N		1411	876	
stunting	Normal	661 (46.8%)	448 (51.1%)	0.093
	Stunted	684 (48.5%)	400 (45.7%)	
		66 (4.7%)	28 (3.2%)	

**Household food security**	**Level**	**Food Secure**	**Mildly Food Insecure Access**	**Moderately Food Insecure Access**	**Severely Food Insecure Access**	**p-value**
N		1744	344	1103	574	
stunting	Normal	1026 (58.8%)	201 (58.4%)	626 (56.8%)	298 (51.9%)	0.005
	Stunted	621 (35.6%)	133 (38.7%)	438 (39.7%)	256 (44.6%)	
	Missing	97 (5.6%)	10 (2.9%)	39 (3.5%)	20 (3.5%)	

**Exclusive Breast feeding**	**Level**	**Non-exclusive BF**	**Exclusively BF**	**p-value**
N		1069	2719	
stunting	Normal	569 (53.2%)	1592 (58.6%)	<0.001
	Stunted	461 (43.1%)	998 (36.7%)	
	Missing	39 (3.6%)	129 (4.7%)	

Supplementary Table 5: Prevalence ratios (95% confidence interval) for the associations of household, maternal and child dietary diversity scores and dietary patterns with childhood stunting in Ethiopia, 2014

	**Prevalence ratio (95% confidence interval)**					**Prevalence ratio (95% confidence interval)**				
	**Household**					**Maternal**				
	**Dietary diversity scores**									
	**<=4**	**>=5**		**P value/ for trend**	**AIC (BIC)**	**<=4**	**>=5**		**P value/ for trend**	**AIC (BIC)**
Model 1	1.00	0.97(0.87-1.09)		0.655	4985 (5107)	1.00	0.93(0.82-1.07)	0.97(0.95-0.99)	0.306	4984 (5107)
Model 2	0.98(0.95-1.01)	-		0.201	4983 (5106)	0.97(0.94-1.01)			0.140	4983 (5105)
	**Dietary patterns**									
	**T1**	**T2**	**T3**			**T1**	**T2**	**T3**		
	**Pattern 1 ("fish, meat and miscellaneous")**					**Pattern 1 ("plant-based")**				
Model 1	1.00	0.99(0.86-1.13)	0.99(0.86-1.13)	0.837	4897 (5026)	1.00	0.93(0.82-1.06)	0.91(0.79-1.05)	0.184	4924 (5052)
Model 2	0.93(0.82-1.06)	-	-	0.299	4894 (5016)	0.79(0.64-0.97)*			0.025	4924 (5046)
	**Pattern 2 ("egg, meat, poultry and legume")**					**Pattern 2 ("egg, meat, poultry and legume")**				
Model 1	1.00	0.97(0.84-1.11)	1.13(0.97-1.30)	0.125	4892 (5021)	1.00	1.13(0.99-1.30)	1.13(0.98-1.30)	0.090	4926 (5054)
Model 2	1.15(0.92-1.43)	-	-	0.217	4894 (5016)	1.16(0.95-1.43)			0.152	4921 (5043)
	**Pattern 3 ("dairy, vegetable and fruit")**					**Pattern 3 ("dairy, vegetable and fruit")**				
Model 1	1.00	0.96(0.84-1.09)	0.83(0.72-0.95)**	0.009	4890 (5018)	1.00	0.94(0.82-1.07)	0.87(0.75-1.01)	0.059	4924 (5053)
Model 2	0.76(0.63-0.93)**	-	-	0.008	4888 (5010)	0.79(0.65-0.95)*			0.015	4920 (5043)
	**Children aged 6-23 months**					**Children aged 24-59 months**				
	**Dietary diversity scores**									
	**<=3**	**>=4**				**<=3**	**>=4**			
Model 1	1.00	1.22(0.87-1.71)	0.92(0.87-0.97)	0.240	1213 (1293)	1.00	0.97(0.85-1.11)	0.99(0.96-1.01)	0.708	3485 (3586)
Model 2	1.06(0.96-1.16)			0.234	1215 (1295)	0.98(0.94-1.03)			0.423	3487 (3589)
	**Dietary patterns**									
	**T1**	**T2**	**T3**			**T1**	**T2**	**T3**		
	**Pattern 1 ("grain-based")**					**Pattern 1 ("grain-based")**				
Model 1	1.00	1.11(0.83-1.49)	1.02(0.79-1.33)	0.880	1218 (1303)	1.00	0.90(0.77-1.04)	0.96(0.81-1.12)	0.508	3488 (3595)
Model 2	1.01(0.95-1.08)			0.690	1216 (1297)	1.00(0.96-1.04)			0.974	3488 (3589)
	**Pattern 2 ("egg, meat, poultry and legume")**					**Pattern 2 ("egg, meat, poultry and legume")**				
Model 1	1.00	0.85(0.65-1.11)	1.03(0.76-1.38)	0.918	1217 (1301)	1.00	1.02(0.87-1.19)	1.07(0.92-1.26)	0.375	3489 (3596)
Model 2	1.05(0.81-1.37)			0.702	1216 (1297)	1.06(0.92-1.23)			0.435	3487 (3589)
	**Pattern 3 ("dairy, vegetable and fruit ")**					**Pattern 3 ("dairy, vegetable and fruit ")**				
Model 1	1.00	0.97(0.75-1.25)	1.03(0.77-1.39)	0.891	1218 (1303)	1.00	1.10(0.94-1.28)	0.86(0.74-1.01)	0.072	3481 (3588)
Model 2	1.02(0.82-1.28)			0.858	1217 (1297)	0.90(0.80-1.01)			0.062	3484 (3586)

	**Children aged 6-59 months**				
	**Dietary patterns**				
	**T1**	**T2**	**T3**		
	**Pattern 1 ("grain-based")**				
Model 1	1.00	0.94(0.82-1.07)	0.97(0.85-1.11)	0.612	4685 (4804)
Model 2	1.00(0.97-1.04)			0.783	4683 (4797)
	**Pattern 2 ("egg, meat, poultry and legume")**				
Model 1	1.00	0.97(0.84-1.11)	1.06(0.92-1.22)	0.457	4683 (4803)
Model 2	1.06(0.93-1.20)			0.410	4683 (4796)
	**Pattern 3 ("dairy, vegetable and fruit ")**				
Model 1	1.00	1.07(0.93-1.22)	0.89(0.78-1.03)	0.138	4679 (4799)
Model 2	0.92(0.83-1.02)			0.116	4681 (4794)

*p<0.05; **p<0.01; AIC – Akaike's information criterion; BIC – Bayesian information criterion

Model 1 was adjusted for both individual (maternal body mass index, age (if applied), household food security, number of under-five children in a household, maternal education, maternal height, exclusive breast feeding) and community-level patterns (water source)

Model 2 was adjusted for all the above covariates but dietary diversity or dietary pattern scores were included as continuous variables without categorization.

P for trend was determined by including the tertiles of dietary patterns as continuous variables (model 1)
